# Application of preoperative 3D printing in the internal fixation of posterior rib fractures with embracing device: a cohort study

**DOI:** 10.1186/s12893-023-02128-x

**Published:** 2023-08-14

**Authors:** Xuetao Zhou, Dongsheng Zhang, Zexin Xie, Yang Yang, Lei Feng, Chunjuan Hou, Menghui Chen, Zheng Liang, Guoliang Zhang, Huiqing Lu

**Affiliations:** Department of Cardiothoracic Surgery, Shijiazhuang Third Hospital, No.15 Tiyu South Street, Chang an District, Shijiazhuang, 050000 Hebei Province China

**Keywords:** 3D printing, Rib fractures, Internal fixation, Total thoracoscopy, Memory alloy embracing device

## Abstract

**Background:**

To explore the impact of preoperative 3D printing on the fixation of posterior rib fractures utilizing a memory alloy embracing device of rib under thoracoscopy.

**Methods:**

The enrolled patients were divided into the 3D printing (11 patients) and the non-3D printing (18 patients) groups, based on whether a 3D model of ribs was prepared prior to surgery. Analysis was conducted comparing the average fixation time per fracture, postoperative fixation loss, and poor reduction of fractured end between the two groups.

**Results:**

The average fixation time of each fracture was 27.2 ± 7.7 min in the 3D printing group and 29.3 ± 8.2 min in the non-3D printing group, with no statistically significant difference observed between the two groups (*P* > 0.05). The incidence of poor fracture fixation in the 3D printing group was statistically lower than that in the non-3D printing group (12.9% vs. 44.7%, *P* < 0.05). Further stratified analysis revealed that the off-plate rate in the 3D printing group and the non-3D group was (3.2% vs. 12.8%, *P* > 0.05), and the dislocation rate of the fractured end was (9.7% vs. 31.9%, *P* < 0.05).

**Conclusions:**

The application of 3D printing technology to prepare the rib model before surgery is proves beneficial in reducing the occurrence of poor fixation of fractures and achieving precise and individualized treatment.

## Background

Rib fractures represent a prevalent type of chest injury [1–3]. Previous treatment modalities, such as compression bandaging, rib traction, and mechanical ventilation, often yielded unsatisfactory outcomes [4]. Consequently, surgical treatment has been explored both domestically and internationally. With the continuous improvement and renewal of medical instruments, internal fixation technology has rapidly developed, and surgical fixation of rib fractures tends to be minimally invasive, which has gained acceptance among medical professionals [5–9]. However, posterior rib fractures, such as those located in the scapular covering and interscapular areas, present complex anatomical structures and pose challenges due to their elevated position and the influence of the scapula. Conventional surgical fixation entails extensive incisions, significant tissue dissection, and often involves pulling the scapula, resulting in considerable damage and aesthetic concerns [10]. With the maturity of thoracoscopic technology and the exploration of minimally invasive surgical approaches for rib fracture fixation, researchers have undertaken studies on transthoracic internal fixation of rib fractures to further mitigate injury [11–13]. Achieving accurate reduction of the fractured ends, optimal selection and molding of fixators, as well as proper placement of fixators during fractured end total thoracoscopic rib fracture fixation. If the fixation shape and placement position are suboptimal during the procedure, it is necessary to repeat the steps of removing the internal fixation, reshaping, and re-placement. The operation is considerably more intricate than the conventional operations leads to prolonged anesthesia and one-lung ventilation. In addition, if the size, positioning, and angle of the fixator do not match the ribs, grave complications may arise post-surgery, such as dislocation of the fractured end, screw detachment, and even the fixator falling into the chest cavity [13]. Hence, preoperative planning and intraoperative reduction of rib fractures constitute pivotal factors in attaining favorable clinical outcomes in the internal fixation of posterior rib fractures, requiring a targeted approach.

Currently, a kind of memory alloy embracing device along with its complementary instruments have been developed in China (Lanzhou Seemine Shape Memory Alloy Co., Ltd) for transpleural thoracoscopic fixation of rib fractures. This embracing device obviates the need for drilling and screw insertion required by locking plates. However, it necessitates meticulous dissection of intercostal tissue and exposure of the rib edge to ensure proper placement of the device, preventing compression of intercostal nerves and blood vessels while enabling comprehensive rib edge embrace. In particular, it is crucial to consider the variability in curvature, longitudinal twist and unrolled curvature of posterior ribs, as well as differences in rib width and thickness, especially at the lower rib margin [14]. Furthermore, the placement of the device at both ends of the fracture and the angle of attachment to the rib merit careful attention. Improper placement can result in poor reduction of the broken rib, disengagement of the device, or failure to fully embrace the rib edges. In addition, the memory alloy embracing device comprises only a pair of embracing arms at both ends. Moreover, the occlusion of the middle junction of the embracing device during placement can make it difficult to identify the fracture line. If the placement position is suboptimal, with the embracing arms is close proximity to the fracture line, the fractured end may detach completely or misalign postoperatively. Thus, precise selection of the appropriate memory alloy embracing device is imperative during the operation.

The utilization of 3D printing has become an integral component of precise surgical interventions spanning various medical domains, playing a pivotal role in the realm of rib fracture surgeries and complex chest wall defects reconstruction [15–17]. In order to address the challenges encountered in thoracoscopic fixation of posterior rib fractures, we reconstructed the 3D rib model according to the preoperative CT scan data, restored the normal shape of the rib model, selected the memory alloy embracing device according to the model, and refined surgical planning. This study aimed to investigate the clinical benefits of accurate preoperative planning and selection of appropriate fixation devices based on 3D rib model in the fixation of posterior rib fractures with embracing device during total thoracoscopic surgery.

## Methods

### Participant data

From September 2019 to July 2022, the cases and data of patients with posterior rib fractures who underwent total thoracoscopic memory alloy embracing device fixation in the Third Hospital of Shijiazhuang were retrospectively analyzed, excluding patients who were lost to follow-up. A total of 29 patients were included, including 11 patients in the 3D printing group and 18 patients in the non-3D printing group. This study was approved by the Medical Ethics Committee of Shijiazhuang third hospital (2021-048), and all patients included in our study provided informed consent.

### 3D printing and preoperative planning

**Fig. 1:**
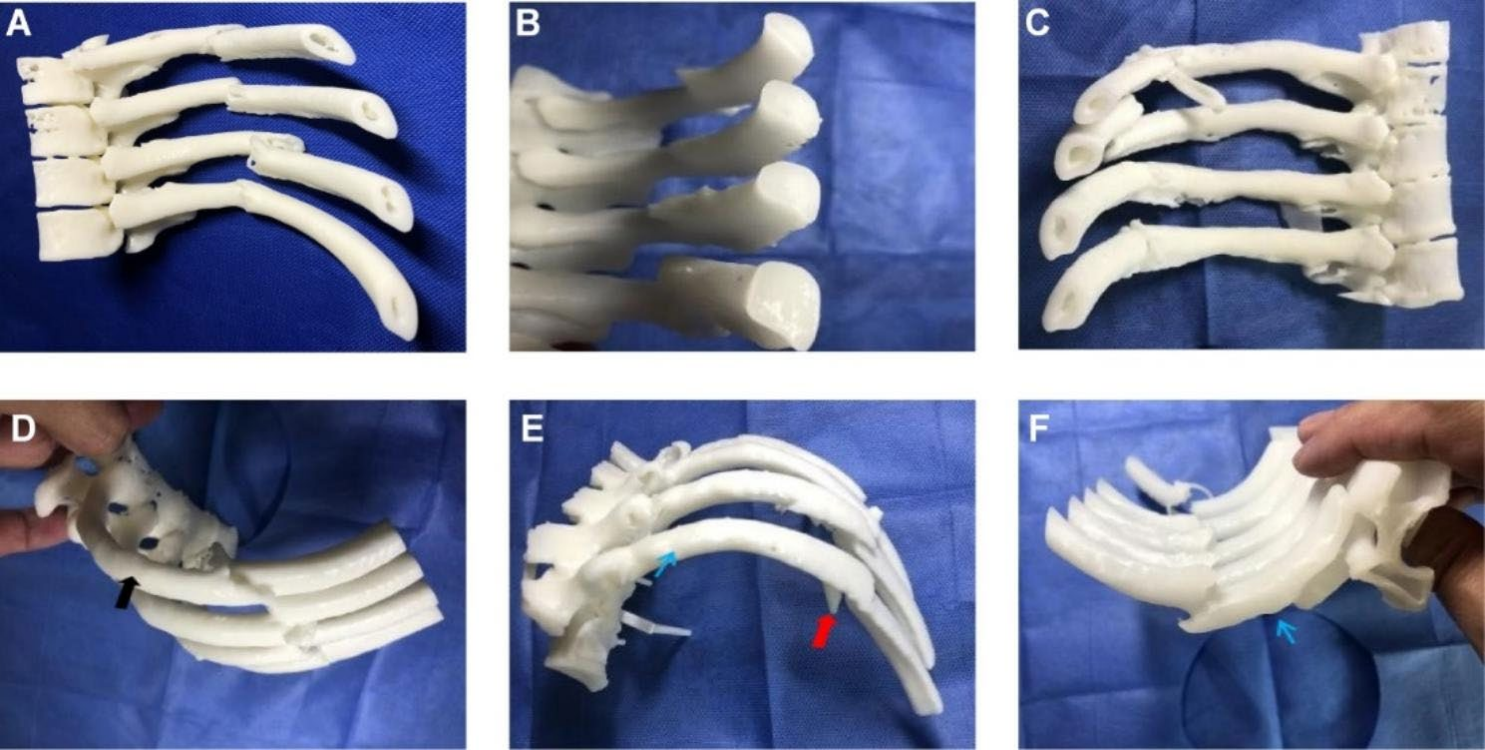
3D printed model of rib fracture made before operation. **(A-C)** The cross section of the ribs shows an irregular triangular shape or water drop shape, with a thick upper margin and thin lower margin. The ribs widen as they move forward. **(D)** Upper margin of ribs broadens. **(E-F)** Lower margin between the transverse process and rib angle thickens. As it moves forward, the lower margin becomes thinner, and a typical rib groove appears

In the 3D printing group, all patients underwent 3D reconstruction using the MDT2AB-010 A software (MDT2AB- 010 A, Meditool Medical Technology (Shanghai) Co., Ltd.) with a thickness of 0.6–1.5 mm of rib CT images before surgery to prepare 3D printing data. The data were imported into the special laser curing 3D printer (pangu4.1, Meditool Medical Technology (Shanghai) Co., Ltd.), and photosensitive resin was used to create a 1:1 3D printed model of the actual rib. The size of the model should include at least 2 cm of length at both ends of the fracture. To maintain the integrity of the fracture state, part of the thoracic vertebra should be included (Fig. 1).

.

**Fig. 2:**
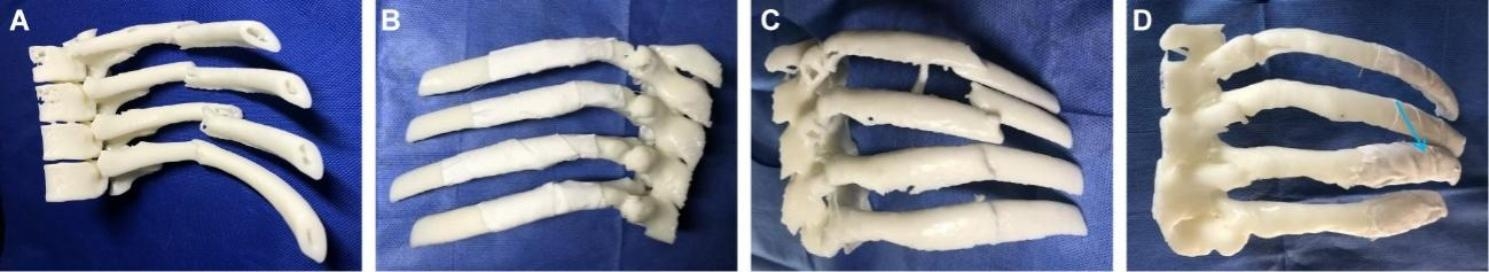
3D model of rib fracture after reduction of fractured end. **(A)** Appearance before reduction. **(B)** Corresponding morphological figure after reset. **(C)** Appearance before reduction. **(D)** Corresponding morphological figure after reset

**Fig. 3:**
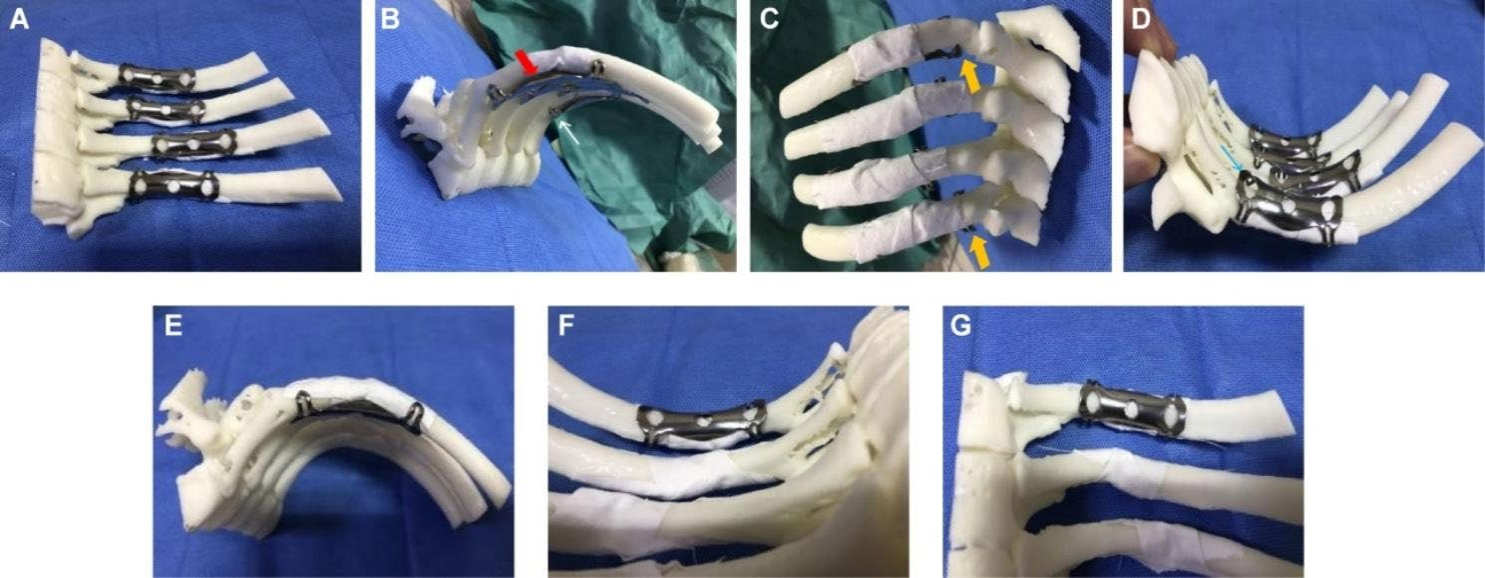
Adjustment of the memory alloy embracing device according to the 3D model. **(A)** Front view showing well-placed hoops. **(B)** Some fixations do not fit well to the ribs (red arrows); an embracing arm fails to wrap around the rib rim, partially off (white arrow). **(C)** Back view showing the embracing device does not match the rib size: the embracing device is wide and the rib is narrow (yellow arrows). **(D)** From the foot side, an embracing arm fails to wrap around rib rim, partially off (blue arrow). **(E-G)** The head side, foot side and front side of the embracing device are well fixed

Detailed surgical planning for each patient was performed according to the 3D printed model of the ribs. The following steps were taken: (1) The morphological characteristics of the fractured ends of each fracture were carefully observed and recorded, especially in cases with small free bone slices, to ensure complete reduction during the operation. (2) The fractured ends of the 3D rib model fracture were reduced and restored to their pre-injury shape (Fig. 2), and the optimal fixation position of the embracing arms of the memory alloy embracing device at both ends of the rib fracture was observed. The structural features of the rib at the site to be fixed were evaluated, taking into account the elliptical and thickest inner rib surface between the transverse process and the rib angle (Fig. 1A-C). As the ribs move forward, the ribs gradually widen and the lower edge becomes thinner, and the cross-section is irregular three-edge shape or water drop shape, and the rib has obvious apparent curvature, longitudinal twist and unrolled curvature (Fig. 1E-F). Therefore, it is necessary to fully evaluate the thickness and width of the ribs and the curvature in all directions according to the rib model to select the most suitable type of memory alloy embracing device. Because of the above multi-angle changes in the ribs, a key step was to evaluate the optimal placement and angle of the two ends of the fracture based on the model after fracture reduction for good attachment. (3) According to the 3D model of fracture reduction, the most suitable embracing device was selected for experimental fixation. The arms at both ends of the embracing device were positioned to completely hug the upper and lower edges of the ribs, and were firmly fixed at both ends of the fracture, so as to avoid the occurrence of various unsatisfactory fixation conditions (Fig. 3). The type of embracing device required for each rib was recorded and send for high temperature and autoclaving for use after planning.

### The technique of fixation of posterior rib fracture with transpleural thoracoscopic memory alloy embracing device

All surgical procedures were performed under general anesthesia with double lumen endotracheal intubation, and prophylactic intravenous antibiotics were administered. The patient was positioned laterally with one-lung ventilation, and a 1.0 cm incision was made at the 7th intercostal incision in the midaxillary line as a 30-cavity endoscopic hole. An incision of approximately 4 cm in length is made in the 3rd or 4th intercostal incisions at the axillary front, and an incision protective sleeve was inserted. The injuries of the thoracic cavity, mediastinum, diaphragm and other tissues were investigated first.

**Fig. 4:**
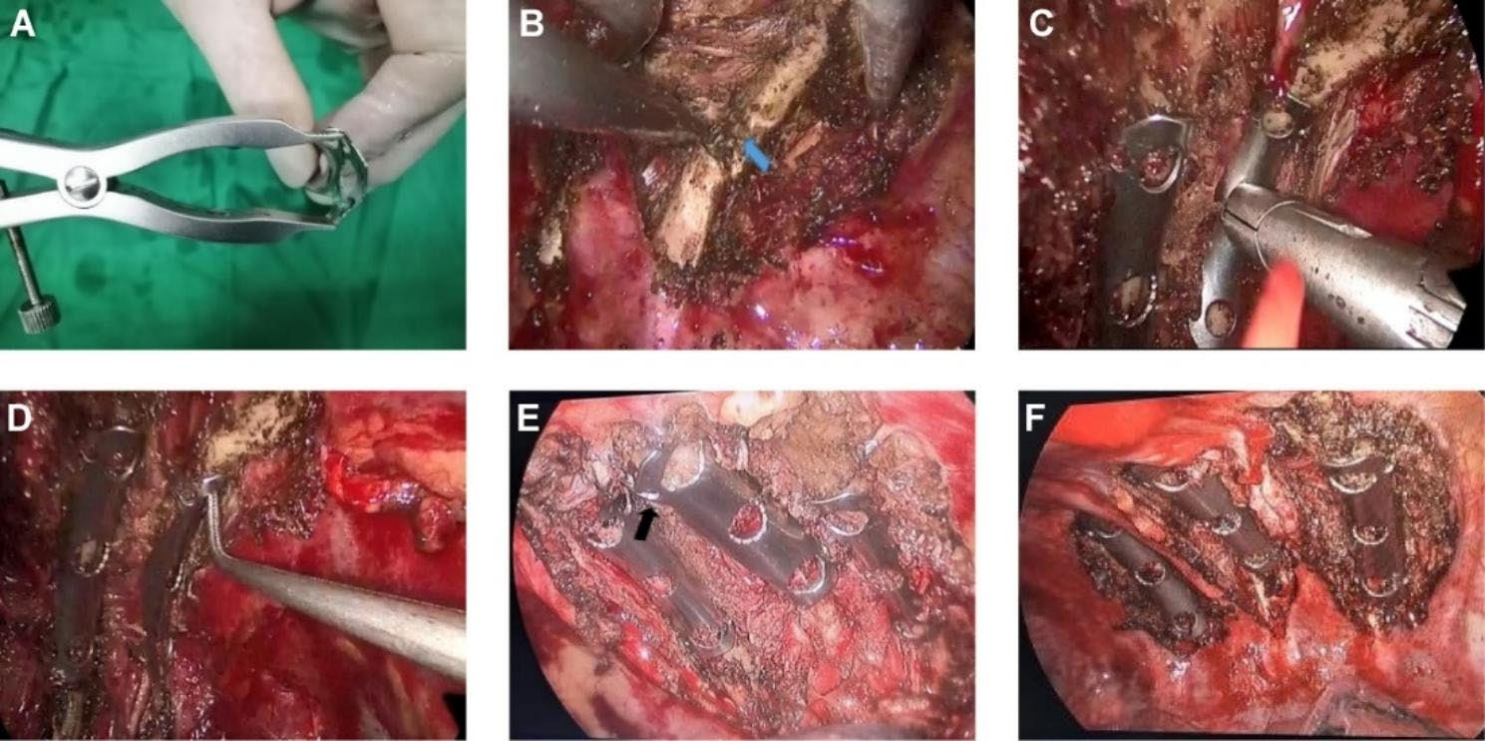
Endoscopic fixation of posterior rib fracture. **(A)** Open the embracing arms. **(B)** Dissect the fractured end. **(C)** Place the embracing device and spray with warm water. **(D)** Test whether the embracing device is stable. **(E)** The embracing device is not properly fixed and partially falls off (black arrow). **(F)** A well-fixed embracing device

According to the characteristics of preoperative CT images and 3D printed rib model, the position of the rib to be fixed and its fractured end were identified under thoracoscopy. The upper and lower edges of the rib at the fracture site were dissociated with an electric hook to fully expose the fractured end of the rib and the upper and lower edges, while protecting the intercostal nerves and blood vessels. The fractured end was then reset using double joint oval forceps and other instruments, depending on the characteristics of the fracture. After pouring 500ml of sterile warm saline into the chest cavity, the memory alloy embracing device was thoroughly cooled for approximately 3–5 min. The selected embracing device was pre-placed in sterile saline at 0–5 ℃ during the dissection of the broken rib. For the ribs to be fixed, the type of the device was selected according to the preoperative 3D printing model. In ice-cold saline, the arms were fully extended with a brace to ensure that the opening was larger than the width of the ribs (Fig. 4A-B). The embracing device was installed on the implantation tool and sent into the chest cavity through the operating port. The position of embracing device was adjusted according to the rib shape, fixed position and attachment angle of the preoperative plan, and the device was gently pushed toward the rib to ensure that each embracing arm crossed the rib edge. The position of the clamp and the embracing device were maintained, and the assistant gently clamped one end of a sterile catheter into the chest cavity with an oval forcep, aligned the device, and sprayed sterile normal saline of about 45℃ to quickly restore it to its original state. The embracing arms were then tightened and fixed at both ends of the fracture. The implantation tool was removed, and the stability of the device was tested by pulling the ring with right angle pliers (Fig. 4C-D).If the position of the embracing device was unsuitable for the curvature or width and length of the rib fracture site, the fractured end was unstable, and the embracing device would had a range of motion (Fig. 4E), it is necessary to apply ice saline gauze cold compress the embracing device for 3–5 min first to soften it and then remove it, so as to prevent further damage. The device was then re-selected the memory alloy embracing device or its placement position was adjusted. The above steps were repeated until the device was firmly fixed (Fig. 4F). When the fixation was completed, hemostasis was fully stopped, a 22 F chest drainage tube was indwelled at the endoscopic observation incision, the lung was fully expanded, and the incision was sutured stratified. The operation was completed, and a chest radiograph or CT scan was performed on the first to third day after the operation. For the non-3D printing group, the position and angle of the memory alloy embracing device could only be adjusted according to the preoperative imaging characteristics and the structural morphology of the ribs after intraoperative reduction.

### Obvervational index

Several variables were collected and analyzed, including the mechanism of chest trauma, age, body mass index (BMI), combined injuries, surgical complications, the number of ribs fixed by memory alloy embracing devices under total thoracoscopy, and the number of poor fracture ends fixation (including fixation loss and poor alignment of fracture end). Patients with other rib fractures are often treated with conventional incision approach surgery or need to undergo chest exploration at the same time, which can significantly increase the total operation time, and the more fixed the rib fractures, the longer the operation time. Therefore, to evaluate the efficiency of surgical fixation accurately, the average fixation time of each fracture in a single patient were calculated. Specifically, the total operation time of the rib fracture fixed by thoracoscopy /the number of fixed fracture ends.

### Statistical analysis

Data collection and preprocessing were performed using an Excel table. Statistical analysis was carried out using SPSS 25.0. Normally distributed variables were described using mean and standard deviation (x ± s), while non-normally distributed data were reported as medians (interquartile range). Two Sample T-tests were used to compare normally distributed variables, while Wilcoxon rank sum tests were used for non-normally distributed data. A p-value of less than 0.05 was considered statistically significant.

## Results

A total of 29 patients were included in this study, 25 males and 4 females, aged 25–73 years (mean 50.1 ± 14.2 years). There were no significant differences in age or BMI between the two groups (Table 1). The main injury mechanism was vehicle accident, accounting for 19 cases, which was the most common injury factor; in addition, heavy object smash injury occurred in 5 cases, falling injury in 4 cases, and crush injury in 1 case. Nineteen patients were complicated with pulmonary contusion and 22 patients were complicated with hemothorax, pneumothorax, hemopneumothorax or other chest injuries. Five patients had splenic contusion,1 patient had renal contusion, and 4 patients had cranial brain injury. Clavicle and scapular fractures were the most common combined fractures. There were 20 patients with unilateral rib fractures and 9 patients with bilateral rib fractures.

**Fig. 5:**
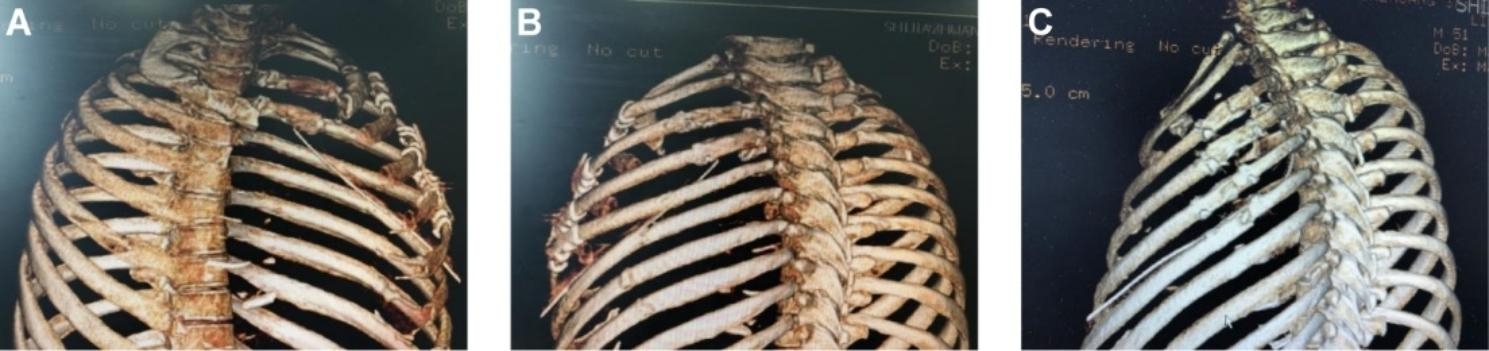
Postoperative CT showing satisfactory fixation with memory alloy embracing device. **(A)** Front view. **(B)** Dorsal view of one patient. **(C)** Dorsal view of another patient

All 29 patients underwent total thoracoscopic fixation of rib fractures with memory alloy embracing devices on the left or right ribs alone. The 3D printing models of the rib fractures to be fixed by VATS were successfully prepared in 11 patients before surgery. A total of 31 fractures were fixed, and the median number of fixed points per person was 3.0 (IQR, 1–5), and the postoperative CT showed memory alloy embracing device fixed satisfactory (Fig. 5). In the non-3D printing group, all 18 patients underwent thoracoscopic fixation of 47 rib fractures with memory alloy embracing devices, and the median number of fixed points per person was 2.0 (IQR, 1–5).

**Fig. 6:**
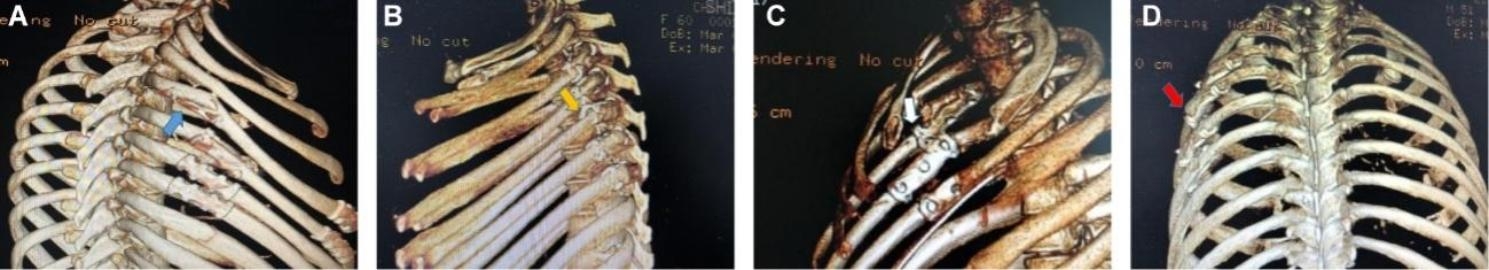
Postoperative CT showing poor fixation with memory alloy embracing device. **(A)** The embracing device failing to adequately embrace the upper and lower edges of the rib and partially detached, causing the fractured end of the fracture to angled downward (blue arrow). **(B)** The embracing device does not match the size of the rib, resulting in unstable and angled downward of the fracture ends (yellow arrow). **(C)** Poor placement of the embracing device, with the front end too close to the fracture line, resulting in unstable fixation and dislocation of the fractured end (white arrow).**(D)** The embracing device is stable, but the fractured end is misaligned backward (red arrow)

As shown in Table 1, the mean fixation time per fracture in the 3D printing group and non-3D printing group was 27.24 ± 7.67 min and 29.27 ± 8.15 min, respectively. Although the average fixation time per site appeared to be shorter in the 3D printing group than in the non-3D printing group, there was no significant difference between the two groups (*P* > 0.05). In the non-3D printing group, 21 out of 47 fixed fractures showed poor fixation of broken ribs, with 6 of the fixations partially detached and 15 of the fractured ends misaligned. Among the total 31 fixations in the 3D printing group, only 4 fractures were poorly fixed, among which 1 fixation partially fell off and 3 fractured ends were misaligned (Fig. 6). Table 1 shown that the rate of poor fracture fixation in the 3D printing group was significantly lower than that in the non-3D printing group (12.9% vs. 44.7%, *P* < 0.05). Further stratified analysis showed that the off-plate rate of the 3D printing group and non-3D printing group was (3.2% vs. 12.8%, *P* > 0.05), and the dislocation rate of the fractured end was (9.7% vs. 31.9%, *P* < 0.05). Although the off-plate rate of the 3D printing group was lower than that of the non-3D printing group, the difference was not statistically significant, while the dislocation rate of the fractured end was statistically significant. There was no complete loss of the memory alloy embracing device in both groups. One patient died of delayed right subclavian artery rupture and hemorrhage caused by trauma 13 days after the operation. Two patients developed pulmonary infections after the operation and recovered after treatment. One patient had wrapped pleural effusion and one patient had venous thrombosis of the lower extremity, which were also cured after treatment.

**Table 1:** Statistical data of patients

Variables	3D group (n = 11)	NON-3D group (n = 18)	Statistic	*P* value
Age (year)	53.2 *±* 14.5	48.4 *±* 13.4	0.91	0.37
ISS	15.8 *±* 4.1	14.8 *±* 5.5	0.89	0.37
BMI (kg/m^2^)	23.6 *±* 3.7	24.1 *±* 5.3	0.29	0.77
Mean fixation time of each rib under endoscope (minute)	27.2 ± 7.7	29.3 ± 8.2	0.70	0.49
The number of fractures fixed under endoscope (IQR)①	3.0 (1, 5)	2.0 (1, 5)	0.42	0.67
Poor fixation rate of fracture end (%)	12.9	44.7	8.66	0.03
Fracture fixation off-plate rate (%)	3.2	12.8	5.20	0.30
Fracture fixation dislocation rate (%)	9.7	31.9	1.08	0.02

IQR: inter-quartile range

## Discussion

The chest represents a highly irregular three-dimensional structure, exhibiting significant anatomical variations in different regions. The rib bone itself is irregular, with differing length, width, and thickness across its different sections, as well as important parameters such as curvature, twist, and unrolled curvature [14]. Despite improvements in clinical selection of rib fracture fixation devices, it remains challenging to fully conform to the anatomical characteristics of ribs. While some researchers have attempted to fix rib fractures through the pleural cavity using thoracoscopic technology, accurate reduction of the fractured end and selection and molding of fixators can be difficult to achieve. The process of precise placement and temporary fixation within the pleural cavity is complex, leading to the design of a memory alloy embracing device and its supporting instruments for transpleural thoracoscopic fixation of rib fractures. In our study, we utilized 3D printing models to gain an intuitive understanding of the three-dimensional shape of the fracture from multiple angles, which enabled us to select an appropriate memory alloy embracing device based on the unique anatomical characteristics of each patient and develop precise surgical plans prior to the operation.

Studies have reported that the potential of 3D printing technology in enhancing preoperative planning, thus improving surgical safety and efficiency [18, 19]. In recent years, there have been studies related to preoperative planning using 3D models in esophagogastric surgery [20], as well as urologic [21] or pediatric cardiothoracic surgery for the treatment of congenital defects [22]. Zeng et al. demonstrated the effectiveness of 3D printing-assisted internal fixation of unstable pelvic fractures via a minimally invasive para-rectus abdominis approach, which facilitated personalized surgical planning, overcame the limitations of poor visual fields in small incisions, and resulted in advantages such as precise operation, minimal trauma, less bleeding, and faster healing [15]. Consistent with previous studies, our results revealed a significantly lower rate of poor fracture fixation and fractured end dislocation in the 3D printing group compared to the non-3D printing group. The observed difference in the dislocation rate of fracture ends between the two groups may be attributed to the complex anatomical structure of the ribs. Firstly, the ribs here are not flat on the inside of the chest. Secondly, from the angle of the ribs to the spine, the thoracic surface is nearly elliptical, with the thickest upper and lower edges. As goes forward, it gradually widens and the lower edge becomes thinner, with a three-edge shape in cross section. And the ribs have obvious apparent curvature、longitudinal twist and unrolled curvature. Thirdly, at present, the type of memory alloy embracing device used under the endoscope is limited, and it is only designed with one kind of radian in the front and back direction, without longitudinal twist. Therefore, it is difficult to determine the optimal adhesion surface to the rib, and the length of the arms at both ends of each memory alloy embracing device is the same and limited, which cannot fully conform to the different changes in the thickness of the ribs. Therefore, it is difficult to select the device according to the experience of the rib shape during the operation and to determine the best position of its attachment to the rib. However, using the 3D rib model before surgery, we were able to observe the changes in the radian, width, and thickness of the fracture site, and select the appropriate memory alloy embracing device accordingly. Additionally, we could fix the device on the 3D rib model to determine the optimal position and angle of attachment between the device and rib. Consequently, the incidence of poor reduction of the fractured end of the fracture was significantly reduced compared to empirical fixation.

Although there was no significant difference in the rate of fixation loss between the two groups, it is worth noting that the 3D printing group exhibited a lower rate of fixation loss compared to the non-3D printing group. Zhou et al. assisted in the preoperative 3D rib model to achieve accurate and individualized treatment of complex rib fractures such as multiple multi-segment rib fractures, high rib fractures, pectoralis major muscle coverage area or scapular coverage area, and shorten the operation time, lowered the difficulty of operation, reduce the injury of patients, and perfect recovery of thoracic shape, the effect is ideal [23, 24]. Moreover, Chen et al. reported that compared with the non-3D printing group, planned surgery with 3D rib model significantly reduced the fixation time of each fracture, shorten the incision length. In addition, under the guidance of 3D model, it is convenient to locate the site of rib fracture and explain the implementation steps of surgical fixation of rib fracture to patients [25]. Overall, these above studies of transthoracic procedures for rib fractures also support our results.

The current literature demonstrates that there are objective benefits to 3D printing in the field of orthopedic surgery, as the utilization of 1:1 scale 3D printed model enables surgeons to gain a comprehensive understanding of the pertinent anatomical structures and their relationships. Consequently, this has resulted in shorter surgical durations and reduced intraoperative blood loss [26–33]. In a study investigating the application of 3D printing technology for precise resection of complex thoracic tumors, 3D printing was employed to preoperatively assess the relationship between the tumor and adjacent organs and blood vessels, aiding in the design of optimal surgical pathways and techniques. The results demonstrated a significant reduction in average surgical time in the 3D printing group compared to the control group (213.2 ± 64.0 vs. 157.7 ± 67.0 min), thus lowering surgical risks [34]. The primary focus of this study was to evaluate the efficiency of surgical fixation, as such, only the average fixation time of each fracture in a single patient were calculated. The results of this study showed that there was no significant difference in the average fixation time per location between the 3D printing group and the non-3D printing group. This finding may be attributed to several factors, such as the relatively small sample size and the complexity of the fixation process. Although the 3D model enables accurate preoperative planning, the placement of the embracing device under total thoracoscopic surgery can still be challenging, often requiring several attempts to achieve satisfactory results. The steps involved in fixing rib fractures with a memory alloy embracing device under endoscopy are also relatively complex and time-consuming. These steps include opening the embracing arm, installing the implantation tool, adjusting the angle of the tool, attaching it to the ribs, and rinsing with warm water to restore the ring arm to its original state. If the memory alloy embracing device is not securely attached to the rib, the fixation may be compromised and require adjustment. This could lead to additional time spent on the operation and potential removal of the device after a few minutes of ice, followed by repeating the aforementioned steps, further affecting the operation time.

In spite of the 3D printing group had the advantage in reducing the incidence of poor fixation, there were also some cases of partial disengagement of the embracing device and poor alignment of the fracture ends in this group. The reasons may be as follows: (1) During the reduction of the broken rib end under thoracoscopy, the overall situation of the alignment of the fractured end could not be visually seen. Because the lateral cortex cannot be exposed, it is not easy to find the deformity of outward angulation. (2) When the fracture ends are long oblique and other unstable fractures, it is difficult to prereset the fracture before placing the embracing device. Often need to use the push force of the device to promote the reduction of the fractured end, the strength is not easy to control and prone to poor reduction of the fractured end. (3) For comminuted fractures, only the pleural surface of the ribs can be separated to reduce and fix the fractured end of the fracture. So, the fragment of the lateral cortex cannot be given anatomic reduction. (4) Anatomically, the ribs are thickest near the thoracic vertebrae and are approximately oval in shape. The anterior part of the rib angle suddenly widened and the lower edge became thinner, and the obvious apparent curvature、longitudinal twist and unrolled curvature of this part changed significantly. At present, the memory alloy embracing device is difficult to meet the above three-dimensional anatomical morphology of the rib and perfectly embrace both ends of the fracture at the same time. Resulting in partial removal of the fixation after surgery. (5) The physical characteristics of 3D printed materials differ from real ribs, which may cause differences in the preoperative experimental fixation of 3D models and intraoperative manipulation of rib fixation. These factors may explain the occurrence of poor fixation in some cases of the 3D printing group.

In view of the many adverse factors of the posterior rib fracture fixed by the memory alloy embracing device in the pleural cavity under thoracoscopic, 3D printing technology was used to make a rib fracture model before surgery, and the surgical plan was precisely planned according to the model, and individualized treatment was realized. The educational role of 3D printing models for novice surgeons is crucial, as it ensures the acquisition of technical skills through the utilization of standardized models [35]. However, there are still some limitations to the application of 3D printing technology in the surgical fixation of rib fractures, such as the time-consuming process, increased cost, and materials that are not conducive to splicing the fractured end. Nevertheless, with the continuous development of 3D printing technology, equipment and materials, the above adverse factors will also be improved. 3D printing technology will become more significant in the transpleural cavity endoscopic memory alloy embracing device fixation of rib fractures. Since most patients in this study underwent non-endoscopic fixation of fractures in other sites, the comparison and evaluation of intraoperative blood loss, postoperative thoracic drainage volume, pain relief, pulmonary function recovery, and complication rates between the two groups were limited. Therefore, future multi-center, large-sample, and randomized controlled studies are needed to further explore the value of 3D printing technology in surgical operations. Despite all this, our findings provide valuable insights into the benefits of using 3D printing technology in medicine and highlight its potential for improving patient outcomes. This study has important implications for the field of medicine and serves as a foundation for future research in this area.

## Data Availability

Data will be made available from corresponding author upon reasonable request.
